# Practical barriers and corresponding solutions in applying interrupted time series to PUMA

**DOI:** 10.1186/1745-6215-16-S2-P135

**Published:** 2015-11-16

**Authors:** Chao Huang, Colin Powell, Davina Allen, Kerry Hood, Emma Thomas-Jones, Nina Jacob, Yvonne Moriarty, Fiona Lugg

**Affiliations:** 1 Cardiff University, Cardiff, UK; 2 Children's Hospital for Wales, Cardiff, UK

## 

Interrupted time series design is an effective quasi-experimental design, which avoids the potential biases in estimating intervention effects by controlling for time series factors (seasonal trend, autocorrelation, etc). It is increasingly adopted in the evaluation of health care interventions, particularly when randomized controlled trials are not feasible. The PUMA (Paediatric early warning system - Utilisation and Mortality) study is a prospective mixed-method before and after study, proposed to investigate the effectiveness of a newly developed Paediatric Early Warning System (PEWS) implementation package. This presentation will discuss some of the practical barriers that were found in applying an interrupted time series approach to this study, such as some key outcomes (such as mortality rate) may have very low event rates (zero values). Also some hospitals are in the process of hospital moving or system switching (from paper-based system to electronic system) during the observation period. We will also discuss the potential statistical solutions that were proposed to overcome these barriers and demonstrate these approaches by the usage of historical cardiac/respiratory arrest data from the University Hospital of Wales with some interesting findings.

